# Gastric Bypass with Long Alimentary Limb or Long Pancreato-Biliary Limb—Long-Term Results on Weight Loss, Resolution of Co-morbidities and Metabolic Parameters

**DOI:** 10.1007/s11695-014-1245-7

**Published:** 2014-04-18

**Authors:** Bent Johnny Nergaard, Björn Geir Leifsson, Jan Hedenbro, Hjörtur Gislason

**Affiliations:** 1 Department of Surgery, Landspitali University Hospital, Reykjavik, Iceland; 2 Department of Surgery, Aleris Hospital, Fredrik Stangs Gate 11-13, 0264 Oslo, Norway; 3 Aleris Obesity Skane, Kristianstad, Sweden

**Keywords:** Bariatric surgery, RYGBP, Weight loss, Measured limbs, Gastric bypass

## Abstract

**Background:**

Several studies indicate that increasing the alimentary limb length in gastric bypass surgery produces only a minor improvement of excess BMI loss. Few studies have addressed the efficacy of increasing the length of the pancreatico-biliary limb.

**Methods:**

Here, we present a prospective randomized study of 187 consecutive laparoscopic Roux-Y gastric bypass operations operated over 2 years (2004–2005) in Iceland. The patients were operated with a gastric bypass with either a 2-m biliopancreatic (BP)-limb and a 60-cm alimentary (A)-limb (*n* = 93) or with a 150-cm A-limb and a 60-cm BP-limb (*n* = 94).

**Results:**

Preoperative median BMI was 44.1 (38–70), median age 35.5 (17–74) years, and 85 % of the patients were female. Follow-up rate after 5 years was 85 %. Eighteen months following surgery, the weight loss was significantly higher in the BP-limb group (*p* < 0.001), and this difference remained 7 years after surgery. Weight regain occurred in both groups, and 7 years after surgery, excess BMI loss (EBMIL) was 78.4 % in the BP-limb group compared to 67.1 % in the A-limb group (*p* < 0.001). Most patients (78 %) needed supplementation adjustment (iron, vitamin D and calcium) during the study period, significantly more often in the BP-limb group compared to the A-limb group (*p* < 0.001). Patients in the BP-limb group had more frequent stools than patients in the A-limb group; otherwise, gastro-intestinal symptoms rating scoring were comparable. Complication rate was similar.

**Conclusions:**

Gastric bypass with a 2-m BP-limb gives better weight loss than gastric bypass with a 60-cm BP-limb and a 150-cm A-limb. Metabolic follow-up is of utmost importance, as most patients needed repeated adjustments of their supplementation.

## Introduction

Mason and Ito introduced gastric bypass (Roux-en-Y gastric bypass (RYGBP)) in the late 1960s, but still, there is no consensus on the ideal length of the gastric bypass limb lengths [1]. The operation results in excess weight loss (EWL) of 70–80 % after 1 to 2 years with high resolution of co-morbidities, but some patients regain significant weight, especially super obese patients. In order to improve results and to find the appropriate balance between benefits and side effects and metabolic sequels, studies with different limb lengths have been performed.

Many studies have been performed with variable lengths of the Roux limb (alimentary (A)-limb), ranging from 45 to 250 cm; they show no or marginal difference in the effect on weight loss. Four of them are randomized studies [2–5], but most are retrospective comparisons [6–10]. Most of these studies are of small volume and not showing data beyond 5 years.

Many authors have demonstrated that weight loss after bariatric surgery and resolution of co-morbidities are strongly associated with a short common channel [11–15], but the shorter the common channel, the more malabsorption and metabolic problems can be expected [7, 12, 14].

However, it should also be noted that no reference can be made to the effect of the ratio of the Roux to the biliopancreatic (BP)-limbs on weight loss and co-morbidity resolution outcomes as there is no available literature that has investigated this issue [16]. Furthermore, long-term weight loss data (>5 years) are not available from randomized controlled trials and are extremely limited in studies of other design [16]. Nevertheless, most authors believe that this ratio is unlikely to have any significant impact on outcome compared to the ratio of the Roux/BP to the common channel length.

**Fig. 1:**
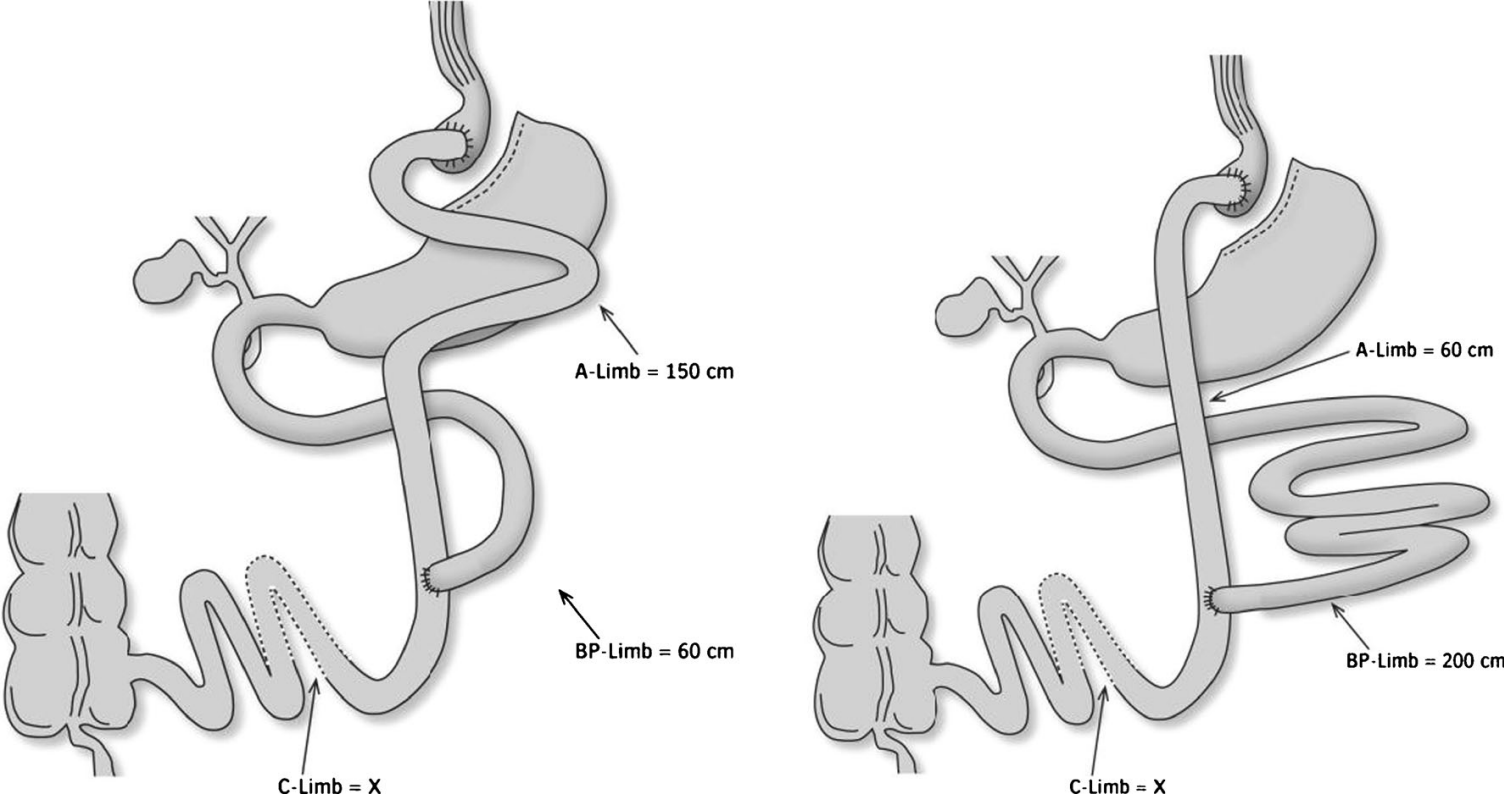
Schematic drawing of the operation with 150-cm A-limb or 200-cm BP-limb

The aim of this study was to compare the effect of the RYGBP with a 200-cm BP-limb (long BP) to a more commonly performed RYGBP with a 150-cm long alimentary limb (150-cm A-limb) and a 60-cm BP-limb (long A) (Fig. 1). The long-term effects on weight loss, resolution of co-morbidities, gastro-intestinal symptoms, metabolic parameters and complications were examined.

## Materials and Methods

### Patients

Prior to this study, our standard laparoscopic gastric bypass procedure was Roux-en-Y gastric bypass with gastric pouch approximately 40 ml, biliopancreatic limb (BP-limb) of 200 cm and alimentary limb (A-limb) of 50 cm [17]. With this procedure, we had a problem with a high incidence of marginal ulcers (16.6 % of patients) [17]. Six (comment 1, reviewer 2) months before the study starts, we changed our procedure to a RYGBP with a gastric pouch of approximately 15 ml, BP-limb of 200 cm and A-limb of 60 cm.

Between January 2004 and December 2005, all 187 morbidly obese patients who underwent primary RYGBP at our institution were included in the study. The same surgical team (HG, BGL) performed all operations. The patient selection criteria were BMI > 40 or BMI > 35 with associated co-morbidity due to obesity (European Guidelines). Prior to surgery, patients participated in a multidisciplinary outpatients’ preparation program, as described in detail earlier [17], and contracted to follow our follow-up program. A protocol was designed at the outset of the study for random allocation of the patients to either of the two arms. The surgical arms are illustrated in Fig. 1. Postoperatively, patients were instructed to take standard supplementation of vitamins and minerals including multivitamin, calcium citrate, vitamin B-12 and vitamin D. Patients were followed up at the outpatient clinic with weight registration and metabolic surveillance, first after 3 months and then every 6 months for 3 years, then once a year.

Resolution of diabetes type 2 was defined as patient without diabetic medication and with glycosylated haemoglobin (HbA1c) within the normal range. Resolution of hypertension was defined as patient without treatment having blood pressure less than 140/90. Patients using continuous positive airway pressure (CPAP) were considered to have sleep apnoea.

Six to 7 years after surgery, patients received a standardized questionnaire including gastro-intestinal symptom rating scale (GSRS). It is an interview-based rating scale consisting of 15 items, originally constructed for the assessment of gastro-intestinal symptoms in the irritable bowel syndrome and peptic ulcer disease [18–21]. For the GSRS, the following five dimensions were used in this study: abdominal pain syndrome (three items), reflux syndrome (two items), indigestion syndrome (four items), diarrhoea syndrome (three items), and constipation syndrome (three items).

This study was approved by the institutional review board and the Icelandic scientific ethical committee VSNa2001050041.

### Statistics

Data from all the patients during the study period were prospectively collected in a proprietary database as part of the hospital records. Proportions are referred to as numbers (%). Continuous data are presented as median and range unless otherwise stated. Differences between proportions were evaluated using Fisher’s exact test. Median differences between groups were evaluated using the Mann-Whitney *U* test. Comparison of mean percentage excess BMI loss (EBMIL%) between groups was done using Student’s *t* test. A *p* value <0.05 was regarded as statistically significant. Weight loss was expressed as percentage excess BMI loss (EBMIL%) plotted against time from operation. The lines were drawn using the locally weighted scatter plot smoothing method (Lowess). All statistical computations were performed using the SPSS version 21.0 for MacOS (SPSS Inc., Chicago).

### Surgical Procedure

Five ports were used: two 5-mm and two 12-mm ports for instruments and one 10-mm port for the camera. A small gastric pouch (15 ml) was created with a 3.5-mm stapler (Endo-GIA II, Tyco/US Surgical). The greater omentum was then split vertically to allow a tension-free antecolic gastro-jejunal anastomosis (GJ anastomosis). The ligament of Treitz was identified, and jejunum measured (loose, not stretched using markers on the graspers) (comment 2, reviewer 2) 60 or 200 cm according to the group included (long A or long BP). The bowel was brought up, first as an omega loop in an antecolic and antegastric fashion, and the GJ anastomosis was created by stapling the jejunum to the posterior wall of the gastric pouch using a linear stapler (3.5-mm stapler). The entero-entero anastomosis (EE anastomosis) was created as a side-to-side anastomosis either 60 or 150 cm below the GJ anastomosis using a 2.5-mm stapler. The remaining openings were hand-sutured and the omega loop divided by the 2.5-mm stapler. The last step was testing the integrity of the gastro-jejunostomy by inflating it with methylene-blue-dyed saline via an NG tube. The mesenteric defects were not closed. Our surgical procedure at the time has previously been described in detail in 2005 [17, 22].

## Results

The results of 187 consecutive patients equally distributed (2–3 patients weekly) over a 2-year period (2004–2005) are presented. Laparoscopic surgery was successfully completed in all cases. Ninety-four patients were randomized to the BP-limb group and 93 patients to the A-limb group. Patient’s characteristics in the two groups are shown in Table 1.

**Table 1:** Patient demographics

	Long BP-limb (*n* = 94)	Long A-limb (*n* = 93)	*p* Value
Age (years)	34 (17–74)	37 (22–61)	0.383
Gender, F/M	79/15 (84 %)	81/12 (87 %)	0.678
BMI	44.5 (39–70)	43.7 (38–68)	0.595
Super obese (BMI > 50)	35 (37.2 %)	31 (33.3 %)	0.647
Weight (kg)	139 (115–225)	134 (92–215)	0.141

Values given are median (range) or %

A total of 85.6 % of patients (160/187) were females, median age 35.5 (17–74). Before the start of the treatment, median weight was 136.5 kg (92–225) and median BMI was 44.1 (38.3–70.3). Sixty-six (35.3 %) patients were super obese (BMI > 50). Mean weight loss prior to surgery was 12.7 kg (0–46), similar in both groups. The median surgical time was 67.3 min (37–112), 68.3 min (40–112) in the BP-limb group compared to 66.3 min (37–94) in the A-limb group (*p* = 0.29) (comment 9, reviewer 2). The median hospital stay was 2.9 days (2–10 days), 2.7 days (2–6) in the BP-limb group and 3.1 days (1–10) in the A-limb group (*p* = 0.30) (comment 9, reviewer 2). There was no difference between the two groups (Table 1).

The diameter of small intestine just below the gastro-intestinal anastomosis was measured in 20 patients in each group with a soft plastic measuring tape (comment 3, reviewer 2). In the A-limb group, the median diameter was 3.5 cm (3.0–4.3) compared to 2.3 cm (1.9–3.1) in the BP-limb group (*p* < 0.05).

**Fig. 2:**
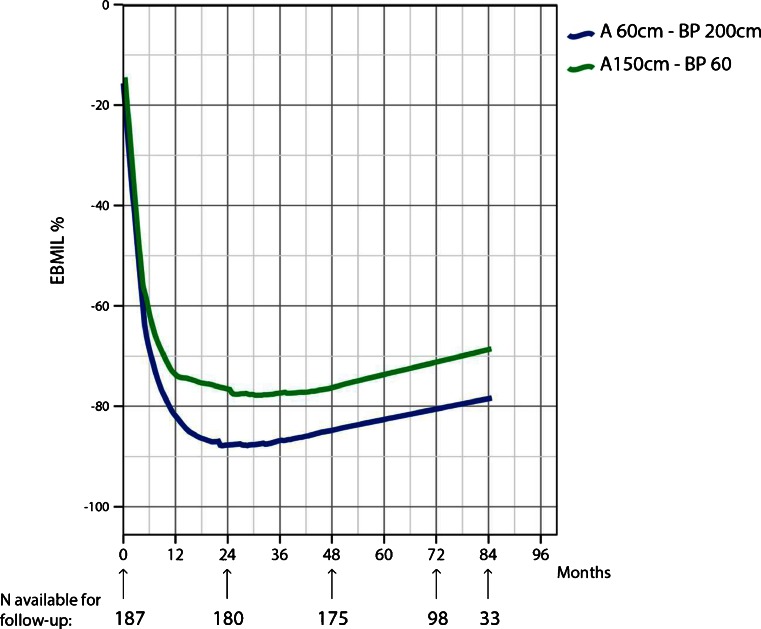
Postoperative weight loss expressed as percentage of excess BMI loss (EBMIL%) plotted against time. The *lines* are drawn using the locally weighted scatter plot smoothing method (Lowess). The figures demonstrate a greater weight loss using the long BP-limb procedure compared to long A-limb procedure

The median follow-up was 70.6 months (0.4–93), similar in both groups. As shown in Fig. 2, 159 patients (85.0 %) attended our outpatient clinic for follow-up of 5 years, and 110 (59 %) completed follow-up 6 to 7 years after surgery. Twenty-eight patients (15 %) had a follow-up of less than 5 years including three patients who died 1, 2 and 4 years after surgery of unrelated causes.

Figure 2 shows the percentage excess BMI loss (EBMIL%) in the two groups. For 18 months, weight loss was significantly higher in the BP-limb group (*p* < 0.001), and this difference remained throughout the study period. Mean excess BMI loss reached a maximum of 88.5 % in the BP-limb group 2 years after surgery compared to 77.7 % in the A-limb group. Weight regain occurred in both groups, and 7 years after surgery, mean EBMIL was 78.4 % in the BP-limb group compared to 67.1 % in the A-limb group. Weight loss failure defined as EBMIL% never exceeding 50 % postoperatively was only seen in four patients in the A-limb group compared to none in the 2-m BP-limb group.

**Fig. 3:**
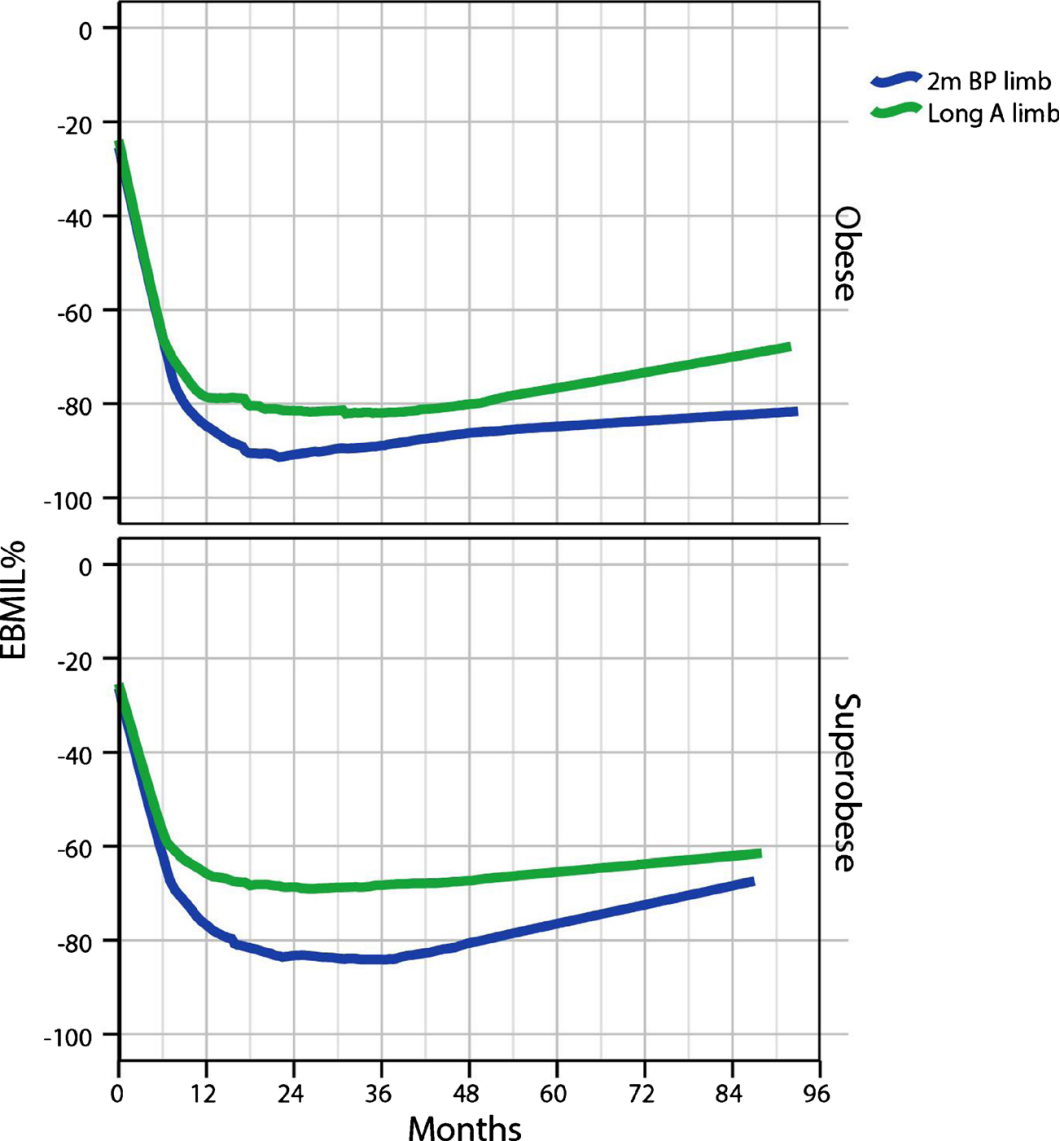
Postoperative weight loss expressed as percentage of excess BMI loss (EBMIL%) plotted against time. The figures demonstrate a greater weight loss in both obese and super obese patients using the long BP-limb procedure compared to long A-limb procedure

Figure 3 (upper part) shows the EBMIL% in those 110 study patients who were not super obese (BMI < 50). Seven years after surgery, mean EBMIL was 82.2 % for the BP-limb group compared to 69.2 % for the A-limb group (*p* < 0.001). Figure 3 (lower part) shows, however, EBMIL% in 66 preoperatively super obese patients (BMI > 50). Seven years after surgery, mean EBMIL was 70.4 % in the BP-limb group compared to 62.8 % in the A-limb group (*p* < 0.01).

Early complications (<30 days) did not differ between the two groups. Two patients had leaks (1.1 %) treated by surgery; five patients (2.7 %) had bleedings, two treated by surgery and three conservatively.

Late complications (>30 days) are shown in Table 2. Seven to 9 years after gastric bypass, 15 patients (8 %) had surgery due to internal hernia, 9 in the BP-limb group and 6 in the A-limb group (*p* = 0.592). Sixteen patients (8.6 %) had marginal ulcers at the gastro-jejunal anastomosis treated conservatively, eight in each group. Twenty-two patients (11.8 %) had surgery due to gallbladder stones (preoperative ultrasound was not performed) (comment 6, reviewer 2).

**Table 2:** Late complications (>30 days)

	All patients (*n* = 187)	Long BP-limb (*n* = 94)	Long A-limb (*n* = 93)	*p* Value
Internal hernia	15 (8 %)	9 (10 %)	6 (6 %)	0.592
Marginal ulcer	16 (8.6 %)	8 (9 %)	8 (9 %)	1.000
Gallbladder stones	22 (11.8 %)	13 (14 %)	9 (10 %)	0.497

Values given are median (range) or %

Co-morbidity was similar in both groups prior to surgery (comment 9, reviewer 2). Table 3 shows the resolution of obesity-related co-morbidities 5 to 9 years after gastric bypass. A total of 20.9 % (39/187) were treated for diabetes type 2 before surgery, 30 with oral antidiabetic medication only and 9 with insulin (comment 7, reviewer 2). Five to 9 years after surgery, 29 (74 %) had resolution of diabetes, no difference between the two study groups. Fifty-nine patients (31 %) were on treatment for hypertension; 39 patients (66 %) had resolution, no difference between the two surgical groups. Prior to surgery, 19 patients had sleep apnoea using CPAP; after 5 to 9 years, 93 % (17/19) were not in need of CPAP. Thirteen patients (6.9 %) had hyperlipidaemia prior to surgery; all of them had a resolution 5 years or more after surgery (comment 10, reviewer 2). Before surgery, 37 patients (19.8 %) had GERD (18 patients in the BP-limb and 19 in the A-limb group) (comment 9, reviewer 2). Joint pain was recorded in 105 patients (56.1 %) prior to surgery (comment 10, reviewer 2). Dramatic improvement of GERD and joint pain was registered in most patients after surgery, similar in both study groups (Table 3).

**Table 3:** Preoperative incidence of obesity-related co-morbidities and the changes 5 to 9 years after Roux-en-y gastric bypass

**A**					
	All patients (*n* = 187)	Study groups	Preoperative incidence (%)	Resolved (%)	*p* Value
Diabetes type 2	39 (20.9 %)	Long BP-limb (*n* = 94)	22 (23)	17 (77)	0.636
		Long A-limb (*n* = 93)	17 (18)	12 (71)	
Hypertension	59 (31.6 %)	Long BP-limb (*n* = 94)	28 (30)	17 (61)	0.406
		Long A-limb (*n* = 93)	31 (33)	22 (71)	
Sleep apnoea	31 (17 %)	Long BP-limb (*n* = 94)	19 (20)	17 (90)	0.245
		Long A-limb (*n* = 93)	12 (13)	12 (100)	
**B**					
		All patients (%)	Long BP-limb (%)	Long A-limb (%)	*p* Value
GERD	Worse	17	8	21	
	No change	4 %	0	10	
	Slightly improved	8	8	10	
	Much improved	8	0	17	
	Resolved	63	84	42	0.140
Joint pain	Worse	19	25	16	
	No change	29	10	41	
	Slightly improved	15	25	9	
	Much improved	29	35	25	
	Resolved	8	5	9	0.136

Values given are number of patients and %; statistic calculated (Chi-square) in each group is based on no improvement versus improvement

Table 4 shows the nutritional parameters in all patients prior to surgery. A total of 35.5 % of the patients had nutritional deficiency before surgery, most commonly vitamin D deficiency (31 %). This was corrected postoperatively by adding supplementation as needed in addition to the standard supplementation.

**Table 4:** Nutritional parameters outside reference values, number of patients (%)

	Before surgery	3 to 9 years after surgery (*n* = 177), median follow-up 71.8 months (36–93)		
	All patients (*n* = 187)	Long BP-limb (*n* = 87)	Long A-limb (*n* = 86)	*p* Value
Albumin	7 (3.7 %)	23 (26.4 %)	18 (20.9 %)	0.394
Vitamin B-12	12 (6.4 %)	24 (26.4 %)	16 (18.6 %)	0.161
Vitamin D	57 (30.5 %)	39 (44.8 %)	18 (20.9 %)	<0.001
PTH	1 (0.5 %)	15 (17.2 %)	3 (3.5 %)	0.003
Iron	2 (6.4 %)	32 (36.8 %)	13 (15.1 %)	<0.001
Ferritin	11 (5.9 %)	58 (66.7 %)	45 (52.3 %)	0.055
Haemoglobin	2 (1.1 %)	53 (60.9 %)	37 (43.0 %)	0.019
Iron and ferritin	0 (0 %)	20 (23.0 %)	12 (14.0 %)	0.126
Iron and ferritin and haemoglobin	0 (0 %)	15 (17.2 %)	10 (11.6 %)	0.185

Values given are number of patients and %; *p* values indicate difference in prevalence between the two arms of the study; *p* < 0.05 is taken to indicate statistical significance

Table 4 shows the percentage of patients in the two groups needing adjustment of supplementation because of their laboratory values being outside the reference values, three or more years after surgery. Most patients (78 %) needed frequent adjustment of their supplementation during the follow-up period of 3 to 9 years. Need for adjustment of iron, vitamin D and calcium citrate was significantly more common in the BP-limb group compared to the A-limb group (*p* < 0.001). Only one patient (long BP) needed hospitalization due to significant but reversible malnutrition problems caused by serious eating disorder. Otherwise, nutrition problems were solved at the outpatient clinic.

A total of 105 patients (56 %) answered the questionnaire. The GSRS scores represented gastro-intestinal symptoms and associated complaints. Higher score represents more symptoms. As shown in Table 5, the diarrhoea score representing frequency of stools, loose stools and urgency was significantly higher in the BP-limb group compared to the A-limb group. The total GSRS score in the BP-limb group was, however, not significantly different from that in the A-limb group (11.1 vs 9.81).

**Table 5:** Assessment of symptoms by the gastro-intestinal symptom rating scale (GSRS)

	Long BP-limb (*n* = 94)	Long A-limb (*n* = 93)	*p* Value
GSRS abdominal pain	2.13 (*n* = *48*)	2.01 (*n* = *57*)	0.60
GSRS constipation	2.09 (*n* = *48*)	2.13 (*n* = *57*)	0.90
GSRS diarrhoea	2.66 (*n* = *48*)	1.19 (*n* = *57*)	0.007
GSRS indigestion	2.76 (*n* = *48*)	2.36 (*n* = *57*)	0.14
GSRS reflux	1.45 (*n* = *48*)	1.40 (*n* = *57*)	0.83
Total GSRS score	11.10	9.81	

Values given are mean (SD); *p* values indicate difference in prevalence between the two arms of the study

## Discussion

The fact that bariatric surgery can fall short of patients’ expectations is troublesome. Both the amount of initial weight loss and the risk of later weight regain are issues that need addressing. The mechanisms behind the effect of gastric bypass are not yet fully understood. We chose to study the effect of different limb lengths in a group of consecutive patients, including both morbidly and super obese.

We found that the longer BP-limb patients did better in terms of weight loss and showed less weight regain over a 7-year period. This effect was achieved at the price of more GI symptoms and a higher need of supplementation.

Why does the 200-cm BP-limb RYGPB results in greater weight loss than the 150-cm A-limb? When performing gastric bypass with a 200-cm BP-limb, the whole jejunum (in most patients) is bypassed and the upper anastomosis is in fact a gastro-ileal anastomosis. The proposed mechanisms could be the following: (a) Food directly to the ileum could affect food tolerance and thereby eating behaviour, and (b) by creating a 200-cm BP-limb, most of the foregut is bypassed altering more, or differently, hormonal and immunological factors. We think the main mechanism is probably the different profile of the GI hormones as demonstrated by numerous recent studies on metabolic surgery (comment 8, reviewer 2). (c) In the 200-cm BP-limb group, 50 cm more of the intestine is bypassed creating consequently a shorter common channel.

In our database, we have measured the total small intestinal length in 650 patients to be 620 cm (420–870 cm) (unpublished results). Thus, the common channel in our study will be more than 3 m in most patients. There is convincing evidence that the degree of malabsorption after gastric bypass is influenced mainly by the length of the common channel rather than the lengths of the Roux or biliopancreatic limbs as bypass is currently constructed by the majority of bariatric surgeons [16]. Thus, the common channel in our study is probably long enough to ensure full uptake of calories, so malabsorption of calories will not explain the difference in weight loss between the two groups. The tendency of the two curves to converge in the super obese group should be interpreted with caution as this is based on few data points. There may also be a follow-up bias favouring those with weight regain problems. Further, follow-up will reveal whether this tendency will hold.

The metabolic problems associated with biliopancreatic diversion and very long limb RYGBP are mostly due to the short common limb [11–15, 23].

We found that 2-m BP-limb is associated with more micronutrient deficiency than 60-cm BP-limb and 150-cm long A-limb, especially iron and calcium deficiency, but these elements are known to be primarily absorbed from the proximal part of the intestines. However, most patients in both groups needed frequent adjustments of their supplementation also 5 years or more after surgery, emphasizing the need for lifelong follow-up after gastric bypass.

Six to 7 years after surgery, gastro-intestinal symptoms were similar in both study groups; the only significant difference was more frequent and looser stools in the BP-limb group. Gastro-intestinal symptoms usually diminish or disappear over time, and differences in symptoms between groups may have been more pronounced earlier after surgery.

Before this study (in our learning curve), we performed a gastric bypass with a 2-m BP-limb and a large gastric pouch (50 ml) [17]; this resulted in high ulcer rate (17 %); the pouch could dilate and many patient had GI symptoms. By reducing the pouch to 15 ml and taking good care of the vascularisation of the major curvature flap, the ulcer rate dropped to 9 % (same in between groups) and the GI symptoms diminished.

Differences in technique other than limb length (such as size of gastric pouch or stoma size) may account for weight loss differences and make it difficult to compare weight loss results between different studies.

Lee et al. [24] performed loop (mini) gastric bypass with the BP-limb tailored according to patients’ BMI from 150 to 350 cm and describe a linear relationship between the reduction in BMI and the BP-limb length. In accordance to our results, the same group found that a routine use of 2-m BP-limb mini bypass not only increased weight loss but also increased the incidence of late nutritional deficiencies, especially anaemia [25].

We conclude that a gastric bypass with a 2-m BP-limb gives a better long-term weight loss than gastric bypass with a 150-cm A-limb, with similar complication rate, but with more nutritional deficiency. This difference is unlikely due to more malabsorption but more likely caused by other mechanisms.
